# Transfering Targeted Maximum Likelihood Estimation for Causal Inference into Sports Science

**DOI:** 10.3390/e24081060

**Published:** 2022-07-31

**Authors:** Talko B. Dijkhuis, Frank J. Blaauw

**Affiliations:** 1 Department of Human Movement Sciences, University of Groningen, A. Deusinglaan 1, 9713 AV Groningen, The Netherlands; 2 Institute of Communication and ICT, Hanze University of Applied Science, Zernikeplein 11, 9747 AS Groningen, The Netherlands; 3 Research and Innovation, Researchable B.V., Office 1.14, Zernikepark 12, 9747 AN Groningen, The Netherlands; f.j.blaauw@researchable.nl

**Keywords:** machine learning, statistics, methods, TMLE, causal inference

## Abstract

Although causal inference has shown great value in estimating effect sizes in, for instance, physics, medical studies, and economics, it is rarely used in sports science. Targeted Maximum Likelihood Estimation (TMLE) is a modern method for performing causal inference. TMLE is forgiving in the misspecification of the causal model and improves the estimation of effect sizes using machine-learning methods. We demonstrate the advantage of TMLE in sports science by comparing the calculated effect size with a Generalized Linear Model (GLM). In this study, we introduce TMLE and provide a roadmap for making causal inference and apply the roadmap along with the methods mentioned above in a simulation study and case study investigating the influence of substitutions on the physical performance of the entire soccer team (i.e., the effect size of substitutions on the total physical performance). We construct a causal model, a misspecified causal model, a simulation dataset, and an observed tracking dataset of individual players from 302 elite soccer matches. The simulation dataset results show that TMLE outperforms GLM in estimating the effect size of the substitutions on the total physical performance. Furthermore, TMLE is most robust against model misspecification in both the simulation and the tracking dataset. However, independent of the method used in the tracking dataset, it was found that substitutes increase the physical performance of the entire soccer team.

## 1. Introduction

Empirical scientific research is intrinsically linked to statistical analysis and modeling. Statistical models are used to better understand phenomena and their underlying *causal* processes that are at play. Researchers rely on empirical data collected from these underlying causal systems that underpin these processes.

In the best case, these data are collected in a controlled environment using a Randomized Controlled Trial design (RCT), a design that has been around for several centuries [1]. However, in many cases, the world is messy, and especially in sports science, an RCT during a match is often impossible, and researchers rely on data obtained from observational studies. While the lack of RCTs seems to make causal inference difficult, methods exist that allow causal reasoning on observational datasets. Furthermore, alternative technologies exist that generally work better than the current status quo [2].

An elite soccer match is inherently only measurable by observing a complex set of latent causal relationships, which complicates the determination of the isolated effects of an event on the outcome. Causal modeling of the influences in a match is intrinsically incomplete and, therefore, applying a statistical method that is mostly robust to incorrectly specified models provide the best understanding of the phenomena. A phenomenon of interest in soccer is the influence of substitutes. In general, substitutions can be initiated by an injury of a player, necessary tactical changes (e.g., because of being behind in a match), or an under-performance of a player [3]. Besides necessary substitutions (e.g., because of an injury), substitution may be the most powerful tool for coaches to influence a match. Substitutions can minimize or offset the effects of fatigue and give new stimuli to the match as elite substitutes introduced during the second half can cover more distance and perform more physically intensive actions relative to whole-match players over the same period [4]. However, the observation that a substitute can cover a greater distance is a fraction of reality [4]. Despite an extensive body of research on substitutes, to the best of our knowledge, there are no studies that investigate the causal effect of the influence of a substitute on the total physical performance of a soccer team. That is: does the total team’s physical performance increase by using substitutes?

One particular field of causal inference that has received traction over the past years is the Targeted Learning approach [5]. The Targeted Learning methodology aims to reconcile traditional statistical inference with modern, state-of-the-art machine learning models. In this paper, we focus our interest on *Targeted Maximum Likelihood Estimation* (TMLE), a method that enables causal reasoning and modeling and that can improve model performance and correctness. TMLE is a semi-parametric double-robust method that can withstand misspecification of the causal model, improving the estimation of effect sizes using machine-learning methods. Double-robust implies that the estimation of the effect remains consistent if either the propensity score model (A propensity score denotes the chance of an treatment given the confounders. If a certain stratum has a higher chance of receiving a treatment (e.g., being female increases the chances of receiving a treatment), a propensity score can be used to control for this.) or the outcome model is misspecified [6].

Although TMLE is not new, its use in the field of sports science is absent. Often traditional methods such as GLMs are used to study the physical performance of teams [7,8,9]. A disadvantage of GLM is that it is not robust in misspecification and is an oversimplified representation of the real world [10]. However, its simplicity is also one of GLMs’ strengths. Assuming the model is well specified, it can give insight into the various essential coefficients for a measured outcome. Such statistical inference is generally impossible to achieve in complicated machine learning models. Machine learning models focus on prediction and learn this by minimizing a loss function instead of focusing on statistical inference [2]. TMLE aims to reconcile statistical inference and machine learning by introducing a two-step approach [2,11,12]. A machine learning algorithm is first trained on the dataset and then adapted to a particular question of interest in the so-called targeting step. With this step, non-parametric models, such as many machine learning models, can be used while statistical inference is still possible [2,13].

The aim of this paper is two-fold. First, we aim to provide a roadmap for making causal inferences in sports science. Secondly, we aim to examine the applicability of the roadmap combined with a study of the performance of TMLE in comparison with the traditional Generalized Linear Model (GLM) in identifying the effect size of a substitute in soccer. On the one hand, we define a simulation study using simulation data on the influence of a substitute on the total soccer team distance as a measure of physical performance. To study the performance of TMLE in comparison with the traditional GLM, the identified substitution effect size of TMLE and GLM are compared using correct and misspecified causal models. On the other hand, we apply observed match data to look at the effect size of a substitute on the total team’s performance in elite soccer using the roadmap combined with TMLE and GLM.

Thus, we provide the basis for bringing causal inference and TMLE into the toolbox of sports science research and improving the quality of causal inference in sports science.

The paper is structured as follows. In Section 2, we present the work that is related to the current study. In this, we focus on scientific literature from the field of substitutes in soccer and from the field of targeted maximum likelihood estimation. In Section 3, we present the methods used in this paper. This section defines the causal roadmap and its application to the current problem. Section 4 presents the results of our study. We present both the results of our simulation study as well as our application of TMLE to substitutions in soccer. Finally, in Section 5 and Section 6, we discuss and conclude the work.

## 2. Related Work

The related work on TMLE and causal modeling and the standard statistical methods to study substitution are the basis for our research on the applicability of causal inference in sports science.

### 2.1. Statistics and Performance of Substitutes in Soccer

Research of performance, substitutes, and soccer, has previously only been performed using traditional statistical methods [3,4,14,15,16]. For example, Bradley, Lago-Penas, and Rey [4] studied the match performances of substitute players using one-way indepent-measures Analysis of Variance (ANOVA). The performance of the substitutes was compared with the players completing the entire match. The meaningfulness of the differences between the substitutes and full match players was indicated by the effect size (ES). Effect size is, as defined by Kelley and Preacher, 2012 [17], “We define effect size as a quantitative reflection of the magnitude of some phenomenon that is used for the purpose of addressing a question of interest”. In the present work, we show, amongst others, that substitutes cover a greater total distance (ES: 0.33–0.67).

Modric et al. [14] investigated the relation between Running Performance (RP) and Game Performance Indicators (GPI). The RP included the total distance covered, distance covered in five speed categories, and the GPI was determined by the position-specific InStat index (InStat, Moscow, Russia). The InStat index is calculated based on a unique set of parameters for each playing position, with a higher numerical value indicating better performance. The exact calculations are only known by the manufacturer of the platform. The associations between RP and GPI were identified by calculating Pearson’s product-moment correlation coefficient. Correlations were found between RP and GPI for different positions. For instance, the total running distance and high-intensity accelerations were correlated with the InStat index for Central Defenders (r=0.42 and r=0.49, respectively).

Hills et al. [3] profiled the match-day physical activities performed by substitutes, focusing separately on the pre- and post-pitch-entry periods. Linear mixed modeling was conducted to differentiate outcome variables as functions of time. A variance components model with no predictors was established for each outcome measure before sequentially allowing intercepts and slopes to vary. A combination of random slopes and intercepts was employed based upon Bayesian information criterion assessments of model fit. One of the conclusions was: substitutes covered a greater (p<0.05) total (+67 to +93 m) and high-speed (+14 to +33 m) distances during the first five minutes of match-play versus all subsequent epochs.

M. Lorenzo et al. [16] aimed, amongst others, to analyze the physical and technical performance of substitute players versus entire-match players or players who were replaced. Linear mixed models analyzed the differences between the performance of substitute, replaced, and entire-match players. Bonferroni’s post-hoc test and Cohens’ *d* conducted the group comparison and the effect size. One of the results was that substitute players showed higher total distance covered (ES: 0.99–1.06), number of sprints (ES: 0.60–0.64), and number of fast runs (ES: 0.83–0.91) relative to playing time than replaced and entire-match players.

All studies mentioned above and their applied methods have in common that they indicate an association between elements of a soccer match but leave out many factors that influence the association’s actual effect size. A combination of the results of Modric et al. [14] and the remaining three [3,4,16] indicate that a substitute player has a better game performance. Even the combination leaves out the influence of the substitutions on the total performance. The methods used and the factors investigated grab only a tiny part of the overall complex system of a soccer match. As Morgulev et al. [18] indicate, it is hard to conclude causality in complex sports systems due to endogeneity problems even when a correlation is found. Endogeneity means either a variable correlated with both the independent variable in the model and with the error term or a left-out variable affecting the independent variable and separately affecting the dependent variable. Complex sports systems are influenced by various left-out factors in the studied phenomenon, making it complex to find causal inference [18].

### 2.2. TMLE and Causal Modeling

Targeted learning is a unique methodology that reconciles advanced machine learning algorithms and semi-parametric inferential statistics [2]. The data available for analysis in sports is proliferating [19] and presents a challenge to both inferential statistics and machine learning. The vast amount of data in sports from, for instance, a semi-automatic multiple-camera video technology in soccer, combined with the inherent complexity of the data-generating process, complicates statistical inference and the underlying mathematical theory. Such as limiting the use of misspecified models, acknowledging that the models do not contain and compensate for the truth, looking for causal relationships in non-experimental data, the proper quantification of uncertainty, etcetera. The challenge is to prevent the specification of uninterpretable coefficients in misspecified parametric models (e.g., GLMs) where different choices of such misspecified models yield different answers [2,20]. In contrast, the targeted learning method (e.g., TMLE) aims to construct confidence intervals for user-specified target parameters by targeting the estimates retrieved from data-adaptive estimators (e.g., machine learning) while relying solely on accurate statistical assumptions. This approach can reduce differences in statistical analysis results as model choices are automated, allowing for consistent estimates regardless of the researcher conducting the study [21].

The Targeted Learning methodology focuses on the art of causal modeling [2]. Causal modeling is a technique used to provide a formal model for and express assumptions about data-generating processes [22,23,24]. Currently, the four main approaches used for causal modeling are (i) Graphical models, (ii) potential-outcome models, (iii) sufficient-component cause models, and (iv) structural equation models [22]. These approaches offer complementary perspectives and can be used together to enhance causal interpretations [25].

With our paper, we aim to introduce a roadmap to use the TMLE methodology in the field of sports science. As such, we introduce causal inference as a new tool in the sports scientists’ toolbox.

## 3. Materials and Methods

We adhere to the *causal roadmap* as a procedure to structure scientific research [22,26]. This roadmap takes the form of seven steps: (i) specifying the knowledge of the system to be studied using a causal model, (ii) specifying the data and their link to the causal model, (iii) specifying the target causality, (iv) assessing identifiability, (v) stating the statistical estimation problem, (vi) estimation, and (vii) interpretation. By following this roadmap, we create a clear distinction between the knowledge about the system under study and about the assumptions that need to be made to answer the research questions; we separate the statistical process from the interpretation process. TMLE is part of this procedure and is applied in the estimation step. The present work adheres to this general structure and is what we see as the blueprint for performing TMLE in sports science.

### 3.1. Specifying the Knowledge of the System to Be Studied Using a Causal Model

**Figure 1:**
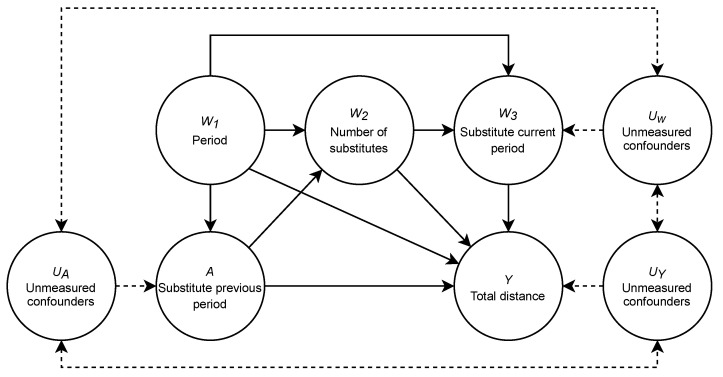
The causal model representation of the system being studied. *Y* = the total distance of a team in five-minute periods; *A* = a substitute or not in the previous five-minute period; $W_{1}$ = the consecutive five-minute periods in the second half of the match (i.e., an index variable indicating the minute of the match); $W_{2}$ = the number of substitutes present; $W_{3}$ = number of substitutes in the current period; *U* = possible unknown confounders influencing *A*, $W_{3}$, and *Y*. The dashed lines indicate that this confounding effect is uncertain.

The first step in this roadmap is to define the knowledge about the system under study. Knowledge, in this case, is actual, fundamental knowledge about the system and should not rely on assumptions of the underlying model. One way to define this system is by using a causal graph representation, which depicts the causal relationships of the system [24]. The causal graph for the influence of a substitute in soccer is shown in Figure 1.

The causal graph shows the causal relationships between variables in the system. For example, an arrow from *A* to *B* describes a causal effect of *A* on *B*, or in other words, *A* causes *B*. This figure also gives rise to some notation that will be used throughout the paper. The nodes on the top of the graph are the *W* variables, which indicate the measured confounders (i.e., factors) in the model, *A* indicates the intervention or treatment that has been performed, *Y* the outcome of the model, and *U* any potential unmeasured confounders that influence our results. With this notation, we aim to stay close to the notation used in other studies (e.g., [2,27]).

#### Case Study

We concretize the aforementioned variables as follows, $W=(W_{1},W_{2},W_{3})$ are the three measured confounders in our model, in which $W_{1}$ is the consecutive five-minute periods in the second half, $W_{2}$ is the number of substitutes present, and $W_{3}$ is whether there was a substitute in the current period. Our treatment variable, A∼B, is a binary intervention that indicates whether a substitution happened in the previous five-minute period. $U_{W,A,Y}∼P_{U}$ are the unmeasured confounders that potentially influence the variables in the model, such as playing home or away, the rank of the teams, the positioning system they play, and the current score. These variables are, by definition, unknown and unmeasured. We do not know whether such variables exist and actually influence the model. However, they could be, which is why they are mentioned here.). $P_{U}$ is the unknown distribution from which $U_{W,A,Y}$ is instantiated. Finally, we have the outcome of our model, Y∼N (in which N denotes the normal distribution), a proxy for performance measured by the total distance covered by the team. A higher distance covered by the team indicates higher performance.

The relationships between these variables are defined as follows; period $W_{1}$ influences the total distance of team *Y*, which is known to decline during the match [4]. As substitutions are highly dependent on the moment of the match; the period $W_{1}$ has a relationship with the substitutes present $W_{2}$, current period substitutions $W_{3}$, and substitutions of the previous period *A*. The total distance of the team *Y* depends on the number of substitutes present given *A* and $W_{2}$, while substitutions cover more distance than entire-match players. When a substitute occurs within the current period $W_{3}$, it leads to a dead ball moment and reduces the overall distance *Y*. Substitutes in the current period and previous period are also influenced by unknown confounders such as an injury or tactical decisions. The overall distance *Y* of a team does not solely depend on the period and substitutes, and other possible unknown confounders *U* in our model are not accounted for but potentially influence the total distance *Y* [28].

After this first step, we have a clear definition of the knowledge and the relationships between the different variables under study, allowing us to move to the data we have about this system.

### 3.2. Specifying the Simulation Data, the Observed Data, and Its Link to the Causal Model

In the second step, we specify the observed and simulation data and its link to the causal model. The causal model we defined in the first step presents *what we know* about the system, whereas the data describes *what we have observed* from it. The causal model describes various possible processes that yielded the data. This description of possible processes is strongly connected to the underlying statistical model of the data; that is, the set of all possible distributions from which the data originates. For this, we define the data as O⊂O∼P, where O is the space of all possible generated data and *P* is the data generating distribution.

#### 3.2.1. Simulation Data

We implemented a data simulator to generate datasets according to the causal model in Figure 1. The code of the data generating system is written in R version 4.0.2 and is available online (https://github.com/dijkhuist/Entropy-TMLE-Substitutions, accessed on 27 July 2022). The observations originating from this simulator are defined as ${\hat{O}}_{i}=\left(W,A,Y\right)∼P_{s},$ in which $W=(W_{1},W_{2},W_{3})$ are the confounders and A∈{0,1} is an indicator variable indicating whether a substitution happened in the previous period. $P_{s}$ is the simulation probability distribution from which the simulation observations $\hat{O}$ were sampled (The hat ($\hat{\;}$) signifies that this is data from the simulator.). The subscript *i* indicates a specific simulation observation ${\hat{O}}_{i}\in \hat{O}$.

#### 3.2.2. Observed Data

We retrospectively collected the in-match position tracking data from 302 competitive professional soccer matches between 18 teams during the Dutch premier league ‘Eredivisie’ 2018–2019 season. The players’ time, position, speed, and acceleration were detected and recorded by the SportsVU optical tracking system (SportsVU, STATS LLC, Chicago, IL, USA). Linke et al. (2018) tested the SportsVU optical tracking system and rated the system as being adequately reliable [29].

For our analysis, two matches with erroneous and missing data were excluded. We only used the second half of the matches, expecting the substitutions to be the most effective. Additionally, the extra time at the end of the second half and goalkeepers were excluded from the dataset. The effect of substitution on the match was controlled by identifying both entire-match players and substitutes. Thus, entire-match players played the entire match, while the substitutes entered the match at a later stage.

**Figure 2:**
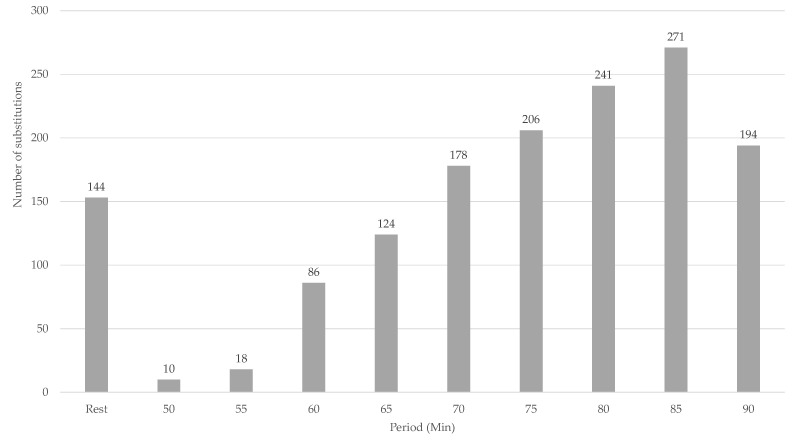
Number of substitutions in the second half per 5-minute period.

**Figure 3:**
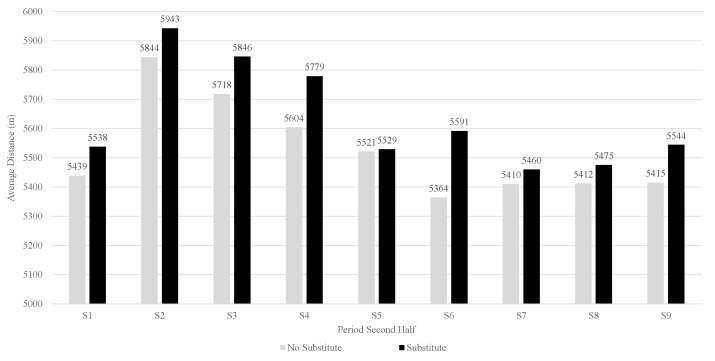
Difference in the total distance when a substitution took place in the previous period or not (*A*).

The dataset was divided into periods of five minutes and consisted of N=5226 observations ($O_{n}$). As an illustration of the data, Figure 2 shows the increasing number of substitutes during the second half. The influence of a substitution in a previous period on the total distance of the team compared to no substitution in the previous period is visualized in Figure 3. Each observation $O_{i}\in O_{n}$ is considered mutually independent (Note that the data we deal with possibly has a stronger dependence than what we are currently showing in our causal model. In fact, *Y* at time *t* could potentially influence $W_{3}$, or even *A* and *Y* itself at time t+1. As our aim with this paper is to introduce TMLE and causal inference in sports, we will not go into detail about the time-dependence of the data. For more information on time series analysis in Targeted Learning, please see [30]). Each of these observations $O_{n}$ is defined as $O_{i}=\left(W,A,Y\right)∼P_{0},$ in which $W=(W_{1},W_{2},W_{3})$ are the confounders, and A∈{0,1} is an indicator variable, indicating whether a substitution happened in the previous period, $P_{0}$ is the unknown real underlying probability distribution from which $O_{n}$ was sampled, and *Y* is the total distance of the team in meters. In the remainder of the work, we will refer to $P_{n}$ as the empirical distribution of the data. The observed dataset is available online (https://github.com/dijkhuist/Entropy-TMLE-Substitutions/tree/main/Data, accessed on 27 July 2022).

Note that in the remainder of the work, we work with a min-max normalized, bounded version of $Y\in \left[0,1\right]$. While this is not relevant for the initial steps of the roadmap, the boundedness of *Y* will become important in the later steps (specifically the estimation step).

### 3.3. Specifying the Target Quantity

The third step in the roadmap is the definition of the target, the causal quantity, or, more specifically, the definition of the causal question of interest. The target quantity can be seen as the main question we would like to answer about the underlying system. Examples of target quantities are: ‘*What is the average treatment effect of a medicine versus placebo?*’ or ‘*How much does gender influence the outcome of a drug?*’. This approach is significantly different from general machine learning approaches, as these generally focus on optimizing a prediction for a multitude number of questions at hand. In contrast, the targeted learning approach only picks one specific question, drastically reducing the complexity of the problem [21]. To define this target quantity, we need to identify the target population with which we are working, the intervention we are doing on this target population, and the outcome we are interested in.

#### Case Study

In our case study, we are interested in determining the effect of substitution (the intervention; *A*) on the total distance in meters (the outcome; *Y*) of the team (the target population). We can further specify our question using the notion of *counterfactuals*; an alternative scenario that has not occurred but that helps us to answer our question. In our case study, we want to see the effect of a substitution A=1 versus not doing a substitution A=0. In some cases, the actual observation we did might not have had a substitution at that time; thus, it represents a ‘counterfactual world.’ Using these counterfactuals, we can adequately define what we are interested in; in our case *we are interested in the difference in team distance between a substitution vs. no substitution simultaneously in time*.

### 3.4. Assessing Identifiability

In the fourth step, we determine identifiability. It should be determined whether sufficient knowledge and data are available to answer the causal question or whether additional assumptions need to be made. The defined causal question can be modeled as an *average intervention effect*, or Average Treatment Effect (ATE) (also referred to as effect size [31]). Formally, an ATE can generally be formulated using the G-computation formula [32],

(1) $$\psi_{0}=\Psi \left(P_{0}\right)=E_{W}\left[E(Y|A=1,W)-E(Y|A=0,W)\right].$$

This G-computation formula determines the average effect of a treatment by determining the average difference between the outcomes for the treated and the non-treated. Note that we use the notation $P_{0}$ here to denote the true probability distribution from which *O* originates (Note that we’re not discussing the unmeasured confounders and the distribution thereof for the sake of clarity. Please see the Targeted Learning book [2] for more details.).

#### Case Study

For the target causality to be identifiable, we need to write our target parameter as a function of the actual distribution $P_{0}$. That is, identifiability would give us $\Psi \left(P_{0}\right)\equiv \Psi \left(P_{n}\right)$. In order to make this claim, we need to impose assumptions on the system. In our case study, we need two assumptions; (i) a positivity assumption and (ii) a no unmeasured confounders assumption (randomization assumption).

The positivity assumption stated as P(A=a∣W)>0∣∀a∈A indicates having enough observations with treatments and controls for all strata of *W*. For each combination of w∈W, we assume that the probability of treatment is greater than zero. If this assumption does not hold, it is not possible to infer the outcomes for the missing strata. The positivity assumption will hold both in the case of simulation data and the observed data (The positivity assumption will not hold when any w∈W is continuous. If that is the case, we need to discretize *W* until the assumption holds.).

The second assumption is the no unmeasured confounders assumption. This assumption states that there is no unmeasured confounding between treatment *A* and outcome *Y*, that is Y⊥⊥A∣W. If we fail to make this assumption, it could be that there is an extraneous variable that influences both our treatment and our outcome variable, yielding the estimation of the causal effect of *A* on *Y* unreliable. In the simulation data, there are no unmeasured confounders, as we control the causal model, the data, and the targeted quantity. This assumption is hard to validate for the observed data, as there are always unmeasured confounders in the real world. As can be seen in Figure 1, we know that there is the possibility that an underlying confounding effect exists, and we assume that, in our case, these effects do not exist/do not significantly impact the outcome of our model. If the dimension of W, measured confounders, is large enough, this assumption is likely to be valid. In the case study, for apparent reasons, this assumption is not satisfied.

### 3.5. Stating the Statistical Estimation Problem

In the fifth step, we state the statistical estimation problem and determine whether all the goals are met to answer our causal question. To perform this estimation, we rely on several assumptions, which are both *knowledge*-based, and *convenience*-based [22]. Knowledge-based assumptions are based on actual knowledge that we have about the causal model and the data. Convenience-based assumptions are assumptions that provide identifiability, if true.

#### Case Study

In our case study (and in many cases), knowledge-based assumptions are not enough to reach identifiability and reason about causality, and as such, we introduced two convenience assumptions; a positivity assumption and an unmeasured confounding assumption (see Section 3.4). These assumptions are needed as we only have limited knowledge about the system we are dealing with. In general, such assumptions should be kept to a minimum (as few as possible, but enough to allow for statistical inference). In our case, the simulation dataset meets both the knowledge-based and the convenience-based assumptions, for we control all aspects of the simulation dataset. In contrast, the tracking dataset meets all assumptions except for the unmeasured confounding assumption.

### 3.6. Estimation

In the sixth step, the actual estimation is performed. Thus far, the roadmap has only helped define the problem we are solving and define the knowledge we have about the problem. With estimation, we aim to find a parameter $\psi_{n}$ as an estimate of the true parameter $\psi_{0}$ of the true data-generating distribution $P_{0}$. To provide some intuition, the observed data, $O∼P_{0}$ is an empirical realization of data retrieved from the true data-generating distribution, $P_{0}$. Suppose $P_{0}$ is controlled by an infinite-dimensional parameter $\psi_{0}$ that controls the data $P_{0}$ generates. Since we do not know $P_{0}$, nor $\psi_{0}$, we aim to find the parameter $\psi_{n}$, which is as close as possible to $\psi_{0}$. We define a mapping function $\Psi :M\overset{}{\to }\psi$, in which M is the statistical model, defining all distributions ($P_{0}\in M$). From this mapping follows that $\Psi \left(P_{0}\right)=\psi_{0}$; that is, the function Ψ yields the true parameter when provided the true distribution. Our goal is to find an estimator based on the empirical data, $\hat{\Psi }\left(P_{n}\right)=\psi_{n}$, in which $\hat{\Psi }:M_{\mathrm{non}-\mathrm{parametric}}\overset{}{\to }\psi$.

To illustrate the process of defining an estimator $\hat{\Psi }\left(P_{n}\right)$ of $\Psi (P_{0})$, our explanation will follow two stages. We will first start with a basic estimation procedure illustrated using a traditional Generalized Linear Model (GLM) approach. Secondly, we show how an estimator of $\Psi (P_{0})$ can be defined using Super Learning and TMLE. We can take this approach as we are dealing with a so-called *substitution estimator* or *plug-in estimator*, allows us to view the implementation of the estimator itself as an implementation detail [2].

#### 3.6.1. GLM-Based Estimation

The general estimation procedure relies on the definition of $Q_{0}$, the relevant part of $P_{0}$ needed for the target parameter. That is, $\Psi \left(P_{0}\right)\equiv \Psi \left(Q_{0}\right)$. In our definition of Ψ in Equation (1), $\Psi (P_{0})$ only relies on ${\bar{Q}}_{0}\left(A,W\right)\equiv E\left[Y|A,W\right]$ and on $Q_{0,W}$, the distribution of *W*. We use the bar (¯) to differentiate between $Q_{0}$ and the element ${\bar{Q}}_{0}$, which is consistent with the other Targeted Learning literature. As such, $Q_{0}$ is defined as the collection $Q_{0}=\left({\bar{Q}}_{0},Q_{0,W}\right)$. With these definitions, we now need to define algorithms that take in the empirical data, and for this, we define the following steps:

1. Estimate ${\bar{Q}}_{0}\left(A,W\right)$ (e.g., using machine learning or a parametric model). That is, build an estimator for E[Y∣A,W].
2. Generate predictions from the estimator for each observation, where we set *A* for each observation (i.e., create counterfactual worlds). That is, we estimate ${\bar{Q}}_{0}\left(A=0,W\right)$ and ${\bar{Q}}_{0}\left(A=1,W\right)$ for each $O_{i}\in O$ (discarding the original values of *A*). With this, we make predictions in the two counterfactual worlds ‘what if everyone received a treatment?’ versus ‘what if no one received treatment?’
3. Estimate $\psi_{n}$ using the G-computation formula as defined in Equation (1).

Note that to estimate $Q_{0,W}$ we use the empirical distribution of *W*, and give each a weight of $\frac{1}{n}$.

In our initial estimation example, we assume a simplistic parametric linear model. Following these steps, we first estimate ${\bar{Q}}_{0}\left(A,W\right)\equiv E\left[Y|A,W\right]$. Using a linear model, such as GLM, this can be estimated as

(2) $${\bar{Q}}_{0,\mathrm{glm}}\left(A,W\right)\equiv E_{n}\left[Y|A,W\right]=\beta_{0}+\beta_{1}A+\beta_{2}W$$

With the formula in Equation (3), we can estimate ${\hat{Y}}_{1}$ and ${\hat{Y}}_{0}$. We use the subscript 1 and 0 on $\hat{Y}$ to indicate that this value of $\hat{Y}$ was calculated by setting A=1 and A=0, respectively. That is, ${\hat{Y}}_{x}$ is the evaluation of Equation (3) for all $O_{n}$, resulting in a list of tuples $\left\{{\hat{Y}}_{1},{\hat{Y}}_{0}\right\}\;\forall \;O_{i}\in O$, which can be used to calculate the ATE as

(3) $$\begin{array}{cc} \psi_{n} & =\frac{1}{n}\sum_{i=1}^{n}\left[E_{n}\left[Y|A=1,W_{i}\right]-E_{n}\left[Y|A=0,W_{i}\right]\right]\\ & =\frac{1}{n}\sum_{i=1}^{n}{\hat{Y}}_{1}-{\hat{Y}}_{0} \end{array}$$

#### 3.6.2. Super Learning and TMLE-Based Estimation

While the linear model provides an initial estimate, the underlying estimator follows a strictly parametric and linear nature, and thus poses various assumptions on the model that we currently cannot assume. To prevent these assumptions, the alternative is to use flexible machine learning techniques in a *super learner* approach, and applying Targeted Maximum Likelihood estimation to perform the estimation of $\psi_{n}$.

Note that we describe some of the background and intuition behind Super Learner and TMLE. For more information and formal proofs, we would like to refer to Van der Laan and Rose [2] (There are also several R packages available that automate the process discussed below. For this, see https://tlverse.org/, accessed on 27 July 2022).

##### Machine Learning and Cross-Validation

Machine learning focuses on the training algorithm to perform an optimal prediction of an outcome *Y* given the input parameters *X*, E(Y∣X). Training a machine learning model works by minimizing a so-called loss function over a series of cross-validation folds.

Cross-validation aims to estimate how well a trained model performs on unseen data by sequentially leaving out data from the training procedure by minimizing a loss function. Cross-validation splits up the data $Z=\{Z_{1},\ldots ,Z_{n}\}$ into training and validation sets. The training and validation sets can be modeled using a random variable $B_{n}\in {\left\{0,1\right\}}^{n}$. With *V* different cross-validation folds, $B_{n}$ can take *V* different values, resulting in a set $b_{1},\ldots ,b_{V}\in {\left\{0,1\right\}}^{n}$. Each $b_{v}$ then corresponds to either of two sets; a training dataset $\left\{Z_{i}:\leq i\leq n,b_{v}\left(i\right)=0\right\}$ or a validation set $\left\{Z_{i}:\leq i\leq n,b_{v}\left(i\right)=1\right\}$. In this case, $b_{v}\left(i\right)$ corresponds to the ith entry of vector $b_{v}$. In our case, we only use one of the splits as a test set, $\sum_{v=1}^{V}b_{v}=1$. Thus, each observation falls once in the validation set and is used V−1 times in the training set.

##### Super Learning

Cross-validation forms the basis of machine learning, and is equally important for super learning. Super learning is a specific instance of machine learning that applies an ensemble methodology to automatically select the best machine learning algorithm or a convex combination of machine learning algorithms. The super learner selects the best estimator among all candidate estimators based on these cross-validation scores [5]. The methodology generally consists of two implementations; the *discrete* super learner and the *continuous* super learner. For each cross-validation fold, the *discrete* super learner starts with a set $L=\{l_{1},\ldots ,l_{m}\}$ learners. These learners can be anything used to perform the prediction E[Y∣X] and could be as simple as a mean of the data and as complex as a neural network or random forest. The super learner trains each $l_{i}\in L$ on each cross-validation fold, resulting in a set of estimators $\bar{L}=\left\{{\bar{l}}_{i,j}\ldots {\bar{l}}_{m,v}\right\}$ and an accompanying cross-validation risk (loss) ${\bar{L}}^{r}=\left\{{\bar{l}}_{i,j}^{r}\ldots {\bar{l}}_{m,v}^{r}\right\}$ for each cross validation fold. Based on these cross-validation risks, the discrete super learner selects the algorithm with the lowest risk by averaging across the folds;

(4) $$\begin{array}{cc} \underset{{\bar{l}}_{m}^{r}\in {\bar{L}}^{r}}{arg min}SL_{d}\left({\bar{l}}_{m}^{r}\right) & =\frac{1}{V}\sum_{j=1}^{V}{\bar{L}}_{m,j}^{r} \end{array}$$

The continuous super learner applies a similar procedure; only instead of selecting the single best estimator, it aims to find weights $\alpha =\{\alpha_{1},\ldots ,\alpha_{m}\}$ where

(5) $$\alpha =\left\{\omega \in R_{+}^{M}:\sum_{m=1}^{M}\omega_{m}=1\right\}$$

for each learner l∈L. The super learner is then defined as the dot product

(6) $$\begin{array}{cc} SL_{c}\left(L,\alpha \right) & =L\cdot \alpha . \end{array}$$

The weights, in this case, are calculated in such a way that they minimize the risk of the $SL_{c}$.

##### Targeted Maximum Likelihood

**Figure 4:**
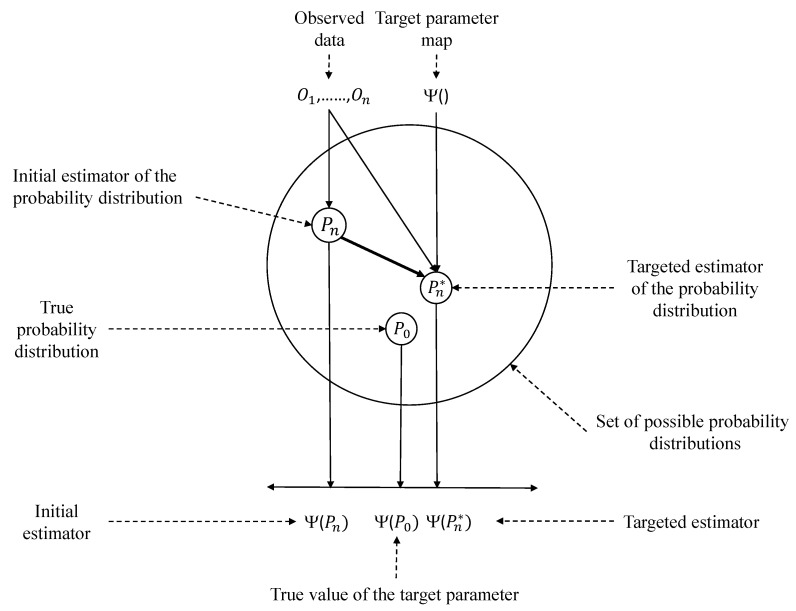
Graphical depiction of TMLE [2].

After the initial estimation step is completed, the next step is to perform the Targeted Maximum Likelihood Estimation (TMLE) step [2,13]. The goal of TMLE is to reduce the bias of the estimation of the target parameter [33]. Figure 4 presents an abstract representation of TMLE and its goal. In this graph, the circle depicts M, the set of all possible probability distributions. As can be seen, $P_{0}\in M$, which maps to the target parameter $\Psi (P_{0})$. Our aim is to use $P_{n}\in M$ with the corresponding $\Psi (P_{n})$ to create $\Psi (P_{n}^{*})$, a targeted estimate closer to the true target parameter.

The definition of the ATE TMLE estimator $\psi^{*}$ is given by

(7) $$\psi^{*}=\frac{1}{n}\sum_{i=1}^{n}\left[{\bar{Q}}_{n}^{*}\left(1,W_{i}\right)-{\bar{Q}}_{n}^{*}\left(0,W_{i}\right)\right].$$

which is the targeted version of ψ (Equation (1)). We use the notation ${\bar{Q}}_{n}^{0}\left(A,W\right)$ to denote the initial estimate of $E\left[Y|A,W\right]$, and ${\bar{Q}}_{n}^{*}\left(A,W\right)$ to denote its targeted counterpart.

Targeting ${\bar{Q}}_{n}^{0}\left(A,W\right)$ involves the two new nuisance parameters; the treatment mechanism $g_{n}\left(A|W\right)$ and the clever covariate $H_{n}\left(A_{i},W_{i}\right)$. The treatment mechanism $g_{n}\left(A|W\right)\equiv P\left(A|W\right)$ can be estimated using, for example, super learning.

The clever covariate can balance the distributions of observed data of the samples under treatment versus the samples under control [11]. The clever covariate is defined for each individual as

(8) $$H_{n}\left(A_{i},W_{i}\right)=\left(\frac{I(A_{i}=1)}{g_{n}\left(A_{i}=1|W_{i}\right)}-\frac{I(A_{i}=0)}{g_{n}\left(A_{i}=0|W_{i}\right)}\right).$$

This clever covariate does not need estimation but is used for fluctuating the initial estimate of ${\bar{Q}}_{n}^{0}\left(A,W\right)$, by relying on information collected about the treatment and control groups (i.e., the ratio between treated vs. control) [11].

Based on these definitions, the steps that are needed in order to estimate the TMLE are as follows (also see the enumeration in Section 3.6):

1. Estimate ${\bar{Q}}_{n}^{0}\left(A,W\right)$ (e.g., using machine learning or a parametric model).
2. Generate predictions from the estimator for each observation, where we set *A* for each observation. That is, we estimate ${\bar{Q}}_{n}^{0}\left(A=0,W\right)$ and ${\bar{Q}}_{n}^{0}\left(A=1,W\right)$ for each $O_{i}\in O$ (discarding the original values of *A*).
3. Estimate the treatment mechanism $g_{n}\left(A|W\right)$.
4. Create the clever covariate $H_{n}\left(A_{i},W_{i}\right)$.
5. Update/fluctuate the initial estimate of ${\bar{Q}}_{n}^{0}\left(A,W\right)$ using the clever covariate.

The last step in this procedure describes updating the initial estimate. This is performed by applying a logistic regression on *Y* on *H*, using our initial estimate as offset. The logistic regression is used to ensure that TMLE is bounded, as introduced by min-max normalizing the outcome variable *Y*. The fluctuation can then be performed on a logistic scale [11].

(9) $$\begin{array}{cc} \mathrm{logit}(E(Y|A,W)) & =\mathrm{logit}\left({\bar{Q}}_{n}^{0}\left(A,W\right)\right)+\epsilon H_{n}\left(A,W\right) \end{array}$$

(10) $$\begin{array}{cc} {\bar{Q}}_{n}^{*}\left(A,W\right) & =\mathrm{expit}\left(\mathrm{logit}\left({\bar{Q}}_{n}^{0}\left(A,W\right)\right)+\epsilon H_{n}\left(A,W\right)\right) \end{array}$$

##### Case Study

For the current simulation study and the case study, we did not implement these steps ourselves but instead relied on existing R-packages that perform most of the calculations. We used the R ‘tmle’ package, version 1.5.0-1 for performing the Targeted Maximum Likelihood Estimation and the ‘superlearner’ R-package, version 2.0-26, for both the simulation study and the case study.

For simulation, we used the data simulation system conforming to the causal model. Because we *know* the exact configuration of this simulator, we can correctly, or purposely incorrectly, specify the data that our learning algorithms take into account. As such, we performed a series of experiments using GLM as defined in Section 3.6.1 and TMLE using super learning, as defined in Section 3.6.2, applying standard learners and handpicked learners (TMLEH): *glm, glm.interaction, step, step.interaction, glm.interaction, gam, randomForest, rpart*. We used the continuous super learner in all experiments. We first calculated the actual expected ATE on the total distance of the soccer team (*Y*) given a substitution in the previous period (*A*) and used that as the ground truth of our simulator. After that, we estimated the ATE of a substitution in the previous period (*a*) on the total distance of the soccer team (*Y*) using the three algorithms mentioned above. First, we used a correctly specified model as input to show the optimal performance of each of the algorithms. After that, we used a misspecified model leaving the substitution of the current period ($W_{3}$) out of the model to indicate how each of the algorithms could cope with this. The code of simulation is written in R 4.0.2 and available online ( https://github.com/dijkhuist/Entropy-TMLE-Substitutions, accessed on 27 July 2022).

Next to the simulation study, we show how TMLE can be applied to the observed dataset. For the application of the observed dataset, we calculated the ATE of a substitution in the previous period (using GLM as defined in Section 3.6.1, TMLE and TMLEH using (continuous) super learning as defined in Section 3.6.2. First, we used a correctly specified model as input to answer the question on the influence of substitution in the previous period (*A*) on the total distance of the soccer team (*Y*). After that, we used a misspecified model leaving the substitution in the current period ($W_{3}$) out of the model to indicate how the algorithms would handle the absence of a confounder. The code of the case study is written in R 4.0.2 and available online (https://github.com/dijkhuist/Entropy-TMLE-Substitutions, accessed on 27 July 2022).

### 3.7. Interpretation

The last step of the roadmap is the estimation interpretation, which depends on the strength of the assumptions made in Section 3.5. The stronger the assumptions, the stronger the relationship between the phenomenon observed and the interpretation. To interpret the results of the data analysis, we can hierarchically depend on the strength of the assumptions on the use of statistical, counterfactual, feasible intervention, or randomized trial [22]. ’The use of a statistical model known to contain the true distribution of the observed data and of an estimator that minimizes bias and provides a valid measure of statistical uncertainty helps to ensure that analyses maintain a valid statistical interpretation. Under additional assumptions, this interpretation can be augmented [22].

#### Case Study

In our case study, we made both knowledge-based and convenience-based assumptions on the simulation dataset and the observed dataset containing the true distribution and allowing the analysis and interpretation to be statistical. Section 4 shows our results and the interpretation thereof.

## 4. Results

**Figure 5:**
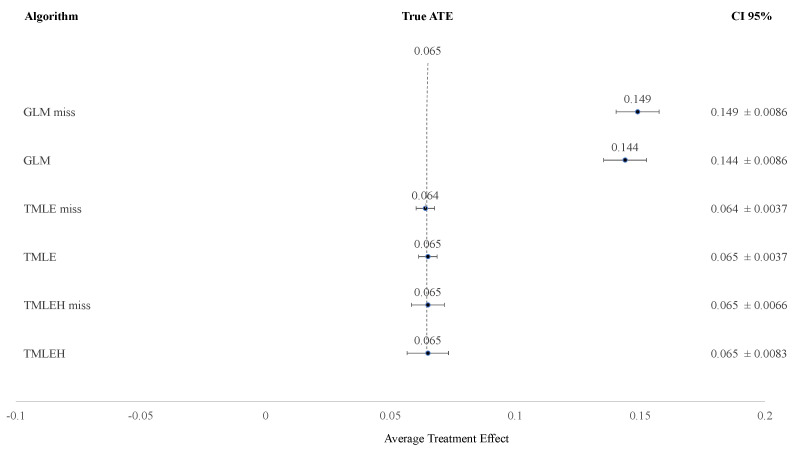
The Average Treatment Effect of the simulation of the causal model and the misspecified causal model. True ATE = True Average Treatment Effect (i.e., effect size) of a substitute in a previous period on the total distance of a soccer team; CI 95% = Confidence Interval 95%; GLM miss = Generalized Linear Model with misspecified causal model; GLM = Generalized Linear Model, TMLE miss = Targeted Maximum Likelihood Estimation with misspecified causal model; TMLE = Targeted Maximum Likelihood Estimation; TMLEH miss = Targeted Maximum Likelihood Estimation using Handpicked algorithms with misspecified causal model; TMLEH = Targeted Maximum Likelihood Estimation using Handpicked algorithms.

Applying the simulation data on the defined causal model, both TMLE and TMLEH have less deviation of the true ATE of the influence of a substitute in the previous period on the total distance of the entire soccer team than GLM (Table 1 and Table 2). When the misspecification of the causal model is applied (e.g., leaving out the substitution in the current period), the increase of deviation of the true ATE is almost non-existent for TMLE and TMLEH, where GLM shows an increased deviation of the true ATE of the influence of a substitute in the previous period on the total distance of the entire soccer team. Figure 5 illustrates the effect of the misspecification, leaving out the substitute of the current period, on the resulting ATE of a substitute in the previous period. Applying the observed dataset, the influence of a substitution in a previous period on the total distance of the soccer team differs per algorithm; the ATE is 0.0105–0.0149 (Table 3). The misspecification of the causal model, leaving out the substitute of the current period, using the real dataset leads to less deviance in TMLE and TMLEH from the respective calculated ATE of the substitute in the previous period on the total distance of the soccer team than GLM (Table 3).

## 5. Discussion

We provided a roadmap as an approach for causal inference. The roadmap was applied to perform causal inference and examine, on the one hand, the performance of TMLE and, on the other hand, the accuracy in estimating the effect size between the traditional method GLM and the novel method TMLE. The comparison between GLM and TMLE was made by performing a simulation study on the effect of substitution on the total physical performance of a soccer team. We showed that GLM yields biased estimates of the effect size, whereas TMLE provides more accurate effect size estimations. These findings are consistent with earlier research [2,11,34].

Furthermore, we applied the causal roadmap using GLM and TMLE on observed elite soccer data. Our results indicate that a substitution in elite soccer increases the total team performance by 0.0105 to 0.01485 of the total distance covered. Other studies on performance, substitutes, and soccer also show that the performance of a substitute is higher when compared to an entire-match player [3,4,16] and that physical performance relates to overall game performance [14]. However, these studies leave out the influence of the substitutions and individual performance on the team’s performance.

The causal roadmap provides a guide for causal inference. It helps to design statistical analyses, answering the causal question while making clear what assumptions are required to provide results with a causal interpretation [35]. Causal inference relates to statistical inference. Where causal inference means reasoning about causation, statistical inference means association reasoning with statistics. Statistical inference aims to assess the parameters of a distribution from samples drawn from that distribution [27]. With the parameters, associations among variables and probabilities of future events can be inferred [27]. The associations and probabilities can be updated when new evidence or new data are available [27]. Causal inference aims to go one step further; the aim is to infer probabilities under static conditions and the dynamics of probabilities under changing conditions, for example, a substitution [27]. That is not to say that statistical inference cannot be used to establish causal relationships. Scientific explanations are an example of applying statistical inference, using, for instance, the Deductive-Nomological Model of Hempel and Oppenheim [36], applying laws to model statistical relevance designed to establish scientific explanations. Scientific explanations are causal explanations establishing a delicate relationship between statistical inference and causal inference. However, causal inference implies the dynamics of changing conditions where statistical inference does not. The combination of the causal roadmap and TMLE offers an opportunity to study the influence of a changing condition.

One limitation of the current study is our application of the causal roadmap. In the first step of this roadmap, it is important to state the knowledge one has about the system under study. The aim of this paper is to introduce readers to TMLE and the causal roadmap. To reduce the complexity of the paper, we have reduced the complexity of the causal model by leaving out some possible time-depending relationships. We believe that this impact is low, but we would advise readers who are dealing with time-series data to look into TMLE methods that make use of time-series data.

TMLE is known as a double robust estimator, meaning that it is consistent whenever the propensity score model is correctly specified, or the outcome regression is correctly specified [6]. Although there are other double robust estimators methods, such as the Augmented Inverse Propensity Weighted (AIWP) Estimator, we limit ourselves to one method.

Van der Laan and Rose [2] compared different methods and found that Maximum likelihood estimation (MLE)-based methods and estimating equations (IPTW and AIPTW) will underperform in comparison with TMLE. Because we aimed to introduce causal inference and targeted learning in sports science, we chose to use the novel TMLE using machine learning and targeted learning.

In our experiments, TMLE and TMLEH outperformed GLM for the observed data between the causal model and the misspecified model. However, the difference in the effect size between the causal model and the misspecified model was considerable for every method. The difference in effect size may be affected by the limited selection of contextual factors. Since well-known contextual factors with an important influence on physical performance, such as match location (home or away), score (win, draw or lose), and rival level [7,8,9], were not available in our dataset and not taken into account. Therefore, our study does not fully meet the second assumption that there is no unmeasured confounding between treatment *A* and outcome *Y*, hence the use of the convenience assumption. In contrast, in the simulation study, we have full control over the data generating distributions and their relationships, and this study, therefore, allows us to fulfill the second assumption. Our goal with the simulation study is to show the applicability of the roadmap and TMLE to a practical problem whilst having an objective means to compare the performance of TMLE to other methods. The double robustness of TMLE implies more resilience to endogeneity, although the double robustness does not solve the endogeneity problem completely. In a study on pharmacoepidemiology, it is found that the more factors are taken into account, the better TMLE performs and becomes more independent of the treatment model specification [12]. When applying the complete set of factors, the outcomes were correct regardless of the treatment model specification [12]. In theory, when all factors are taken into account in the performance of a soccer team, TMLE will engage the true influence of a substitution.

## 6. Conclusions

Our study set out to provide a roadmap for causal inference and introduce the use of TMLE in sports science for other sports scientists. We applied the causal roadmap and showed that TMLE has a lower bias than GLM in a simulation setting both on the correct and the misspecified causal model. This result indicates that TMLE can be a more precise method than GLM in identifying and correctly estimating causal effects. Furthermore, when applying GLM and TMLE to the observed data on substitutions, both methods found that the total physical performance improves when a substitution is made. However, the difference in the effect sizes between the correctly specified and the misspecified model was considerable for TMLE and GLM. Furthermore, we showed that in these cases, TMLE was more precise than GLM.

## 7. Practical Implications

These findings show that the power of TMLE can help bring causal inference in sports science to the next level when more factors are taken into account. Future work will need to collect as much factor data as possible, enabling investigation of the influence of one factor in contrast with the traditional statistical methods where a selection of factors is made.

## Figures and Tables

**Table 1:** Simulation of the correct causal model.

True ATE: 0.0646			
Measure	GLM	TMLE	TMLEH
ATE	0.1442	0.0647	0.0647
Confidence Interval 95%	0.1399–0.1485	0.0628–0.0665	0.0605–0.0688
Bias	0.0797	0.0001	0.0001
Bias %	123.50	0.22	0.17

GLM = Generalized Linear Model; TMLE = Targeted Maximum Likelihood Estimation; TMLEH = Targeted Maximum Likelihood Estimation using Handpicked algorithms; ATE = Average Treatment Effect (i.e., effect size) of a substitute in a previous period on the total distance of a soccer team.

**Table 2:** Simulation of misspecified causal model.

True ATE: 0.0646			
Measure	GLM	TMLE	TMLEH
ATE	0.1491	0.0647	0.0646
Confidence Interval 95%	0.1399–0.1485	0.0628–0.0665	0.0613–0.0679
Bias	0.0846	0.0001	0.0000
Bias %	131.00	0.22	0.00

GLM = Generalized Linear Model; TMLE = Targeted Maximum Likelihood Estimation; TMLEH = Targeted Maximum Likelihood Estimation using Handpicked algorithms; ATE = Average Treatment Effect (i.e., effect size) of a substitute in a previous period on the total distance of a soccer team.

**Table 3:** Observed dataset causal model.

Measure	GLM	TMLE	TMLEH
**Correct causal model**			
ATE	0.0105	0.0149	0.0142
Confidence Interval 95%	–0.0007–0.0216	0.0007–0.0290	–0.0021–0.0303
**misspecified causal model**			
ATE	0.0193	0.0245	0.0247
Confidence Interval 95%	–0.0007–0.0216	0.0115–0.0374	0.0210–0.0381
**Difference correct causal model and misspecified causal model**			
Difference correct causal model and misspecified	0.0089	0.0096	0.0121
Difference correct causal model and misspecified %	84.7	65.0	66.3

GLM = Generalized Linear Model; TMLE = Targeted Maximum Likelihood Estimation; TMLEH = Targeted Maximum Likelihood Estimation using Handpicked algorithms; ATE = Average Treatment Effect (i.e., effect size) of a substitute in a previous period on the total distance of a soccer team.

## Data Availability

The data can be found on Github: https://github.com/dijkhuist/Entropy-TMLE-Substitutions/tree/main/Data (accessed on 27 July 2022).
